# Induction of Heme Oxygenase-1, Biliverdin Reductase and H-Ferritin in Lung Macrophage in Smokers with Primary Spontaneous Pneumothorax: Role of HIF-1α

**DOI:** 10.1371/journal.pone.0010886

**Published:** 2010-05-28

**Authors:** Delphine Goven, Anne Boutten, Véronique Leçon-Malas, Joëlle Marchal-Sommé, Paul Soler, Jorge Boczkowski, Marcel Bonay

**Affiliations:** 1 Inserm, U700, Faculté de Médecine-Site Bichat, Université Denis Diderot-Paris 7, Paris, France; 2 Services de Biochimie A, Hôpital Bichat, Assistance Publique-Hôpitaux de Paris (AP-HP), Paris, France; 3 Centre d'Investigation Clinique 007, Paris, France; 4 Inserm, U955, Faculté de Médecine, Groupe Hospitalier Universitaire Albert Chenevier - Henri Mondor, Créteil, France; 5 Faculté de Médecine, Université Paris 12, Créteil, France; 6 Service de Physiologie-Explorations Fonctionnelles, Hôpital Bichat, Assistance Publique-Hôpitaux de Paris (AP-HP), Paris, France; Emory University, United States of America

## Abstract

**Background:**

Few data concern the pathophysiology of primary spontaneous pneumothorax (PSP), which is associated with alveolar hypoxia/reoxygenation. This study tested the hypothesis that PSP is associated with oxidative stress in lung macrophages. We analysed expression of the oxidative stress marker 4-HNE; the antioxidant and anti-inflammatory proteins heme oxygenase-1 (HO-1), biliverdin reductase (BVR) and heavy chain of ferritin (H-ferritin); and the transcription factors controlling their expression Nrf2 and HIF-1α, in lung samples from smoker and nonsmoker patients with PSP (PSP-S and PSP-NS), cigarette smoke being a risk factor of recurrence of the disease.

**Methodology/Principal Findings:**

mRNA was assessed by RT-PCR and proteins by western blot, immunohistochemistry and confocal laser analysis. 4-HNE, HO-1, BVR and H-ferritin were increased in macrophages from PSP-S as compared to PSP-NS and controls (C). HO-1 increase was associated with increased expression of HIF-1α mRNA and protein in alveolar macrophages in PSP-S patients, whereas Nrf2 was not modified. To understand the regulation of HO-1, BVR and H-ferritin, THP-1 macrophages were exposed to conditions mimicking conditions in C, PSP-S and PSP-NS patients: cigarette smoke condensate (CS) or air exposure followed or not by hypoxia/reoxygenation. Silencing RNA experiments confirmed that HIF-1α nuclear translocation was responsible for HO-1, BVR and H-ferritin induction mediated by CS and hypoxia/reoxygenation.

**Conclusions/Significance:**

PSP in smokers is associated with lung macrophage oxidative stress. The response to this condition involves HIF-1α-mediated induction of HO-1, BVR and H-ferritin.

## Introduction

Primary spontaneous pneumothorax (PSP), representing a medical emergency, is an important health problem. PSP is defined as air occurrence in the pleural space with secondary lung collapse atelectasis. This disease is common in young, otherwise healthy people and is frequent in clinical practice (1%-7% of all pulmonary diseases). Recurrence of pneumothorax is a frequent phenomenon (approximately 30%) and a critical problem in disease management [1], [2]. Cigarette smoking is suggested as a risk factor for PSP [3] and is an independent risk factor for recurrence [4].

Few data are available concerning the pathophysiology of PSP and the role of cigarette smoke in this process. De Smedt and coworkers [5] showed inflammatory changes in the bronchi, lung parenchyma and pleural cavity accompanied by anatomical and histological changes in bronchi and lungs. Oxidative stress could be involved in the predisposing effects of cigarette smoking because cigarette smoke contains a high oxidative burden [6]. Indeed, oxidative stress has been implicated in the pathogenesis of many respiratory diseases, especially those induced by cigarette smoke [7], [8]. In these pulmonary diseases, macrophages play a crucial role in the oxidative stress response [9], [10]. However, no information is available on oxidative stress in lung macrophages of patients with PSP and its relation with smoking status.

Heme oxygenase (HO) plays a critical role counterbalancing oxidative stress. HO catalyses heme degradation producing CO, which has anti-inflammatory properties; biliverdin converted to bilirubin, a powerful antioxidant, by biliverdin reductase (BVR); and free iron bound to the heavy chain ferritin (H-ferritin), another antioxidant molecule. In the lung, the inducible, redox-sensitive, isoform of HO (HO-1) is involved in protection against inflammation and oxidative stress in different pathological conditions [11]. Therefore, HO-1 could be induced in the lung of patients with PSP, especially smokers, to provide protection against inflammation and oxidative stress. However, to the best of our knowledge, no data is available on oxidative stress markers and HO-1 expression in the lungs of patients with PSP, whatever their smoking status. Therefore, we first aimed to investigate the mRNA and protein expression of HO-1, BVR and H-ferritin, along with 4-hydroxynonenal (4-HNE) adducts, a marker of oxidative stress, in lung biopsies of smoker and non-smoker patients with and without recurrent PSP disease.

During the pneumothorax condition and treatment, atelectasis and reexpansion are associated with acute alveolar hypoxia and reoxygenation, respectively [12]. Therefore, our second aim was to examine the mechanism(s) involved in inducing HO-1, BVR and H-ferritin expression in PSP by analyzing the expression of and the roles of transcription factors involved in signaling events after hypoxia/reoxygenation and/or cigarette smoke exposure in the aforementioned lung biopsies and *in vitro* in a human monocyte/macrophage cell line (THP-1). We focused on hypoxia inducible factor 1α (HIF-1α) and nuclear factor erythroid 2-related factor 2 (Nrf2) [13], [14].

## Results

### Characteristics of lung samples

**Figure 1:**
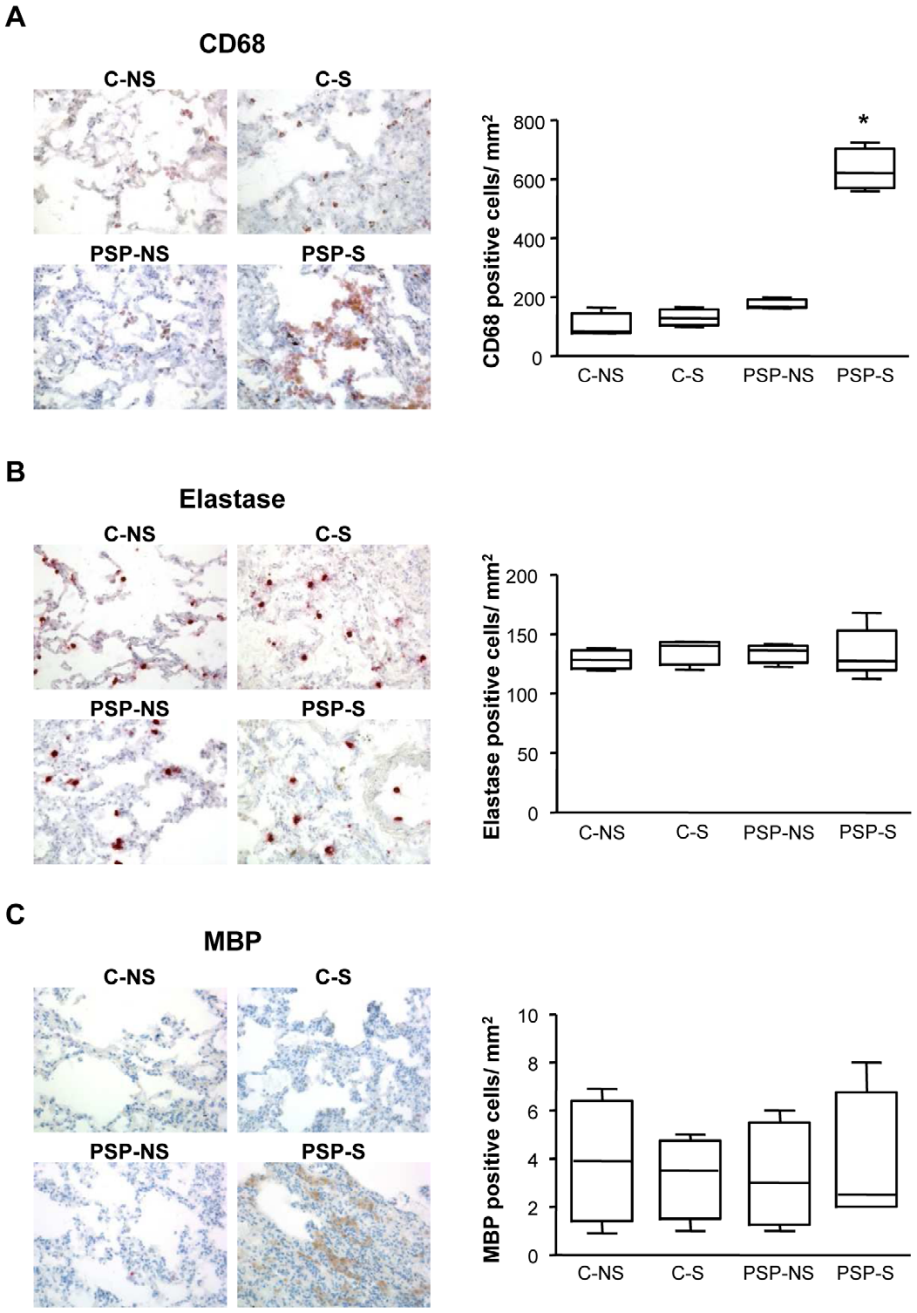
Detection of CD68, elastase and major basic protein (MBP) in lung biopsies. Specific immunohistochemical staining and quantification of CD68 (A), elastase (B), MBP (C) were assessed in lung biopsies from C-NS, C-S, PSP-NS and PSP-S patients (magnification ×200). C-NS and C-S, control patient nonsmokers and smokers, respectively; PSP-NS and PSP-S, primary spontaneous pneumothorax nonsmokers and smokers, respectively. Box-and-whiskers plot with median, interquartile range and minimum and maximum values. *p = 0.029 for CD68, PSP-S vs. PSP-NS, C-NS and C-S. Images are representative of all samples.

Histological analysis of lung biopsies showed inconstant subpleural areas of fibrosis with adjacent subpleural bullae in primary spontaneous pneumothorax patients (PSP), and no alterations in control patients (C). The number of macrophages was significantly higher for primary spontaneous pneumothorax smokers (PSP-S) than primary spontaneous pneumothorax nonsmokers (PSP-NS) and C patients (p = 0.029; Figure 1A). The groups did not differ in polymorphonuclear neutrophils (p = 0.62) or eosinophils number (p = 0.97) (Figure 1B and 1C).

### Oxidative stress in PSP-S samples

**Figure 2:**
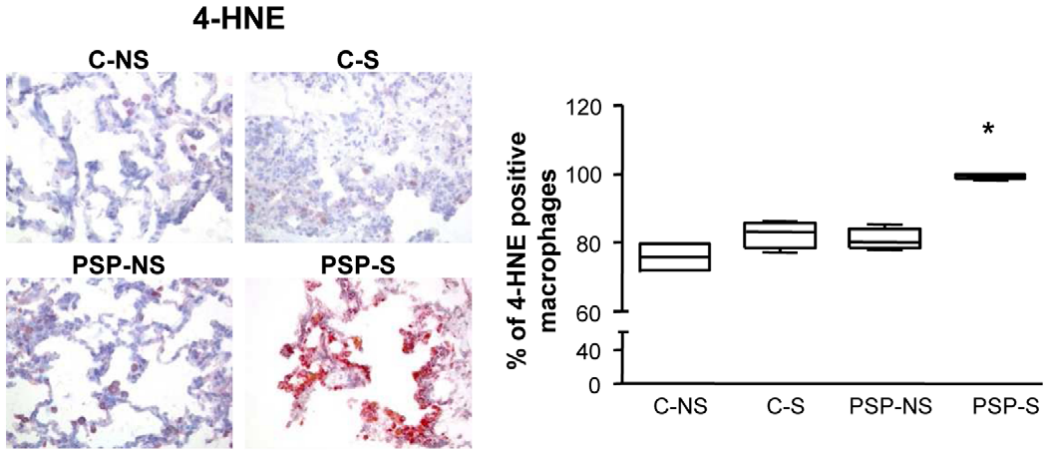
Oxidative stress in lung biopsies. Specific immunohistochemical staining and quantification of 4-HNE (a marker of oxidative stress) were assessed in lung biopsies from C-NS, C-S, PSP-NS and PSP-S patients (magnification ×200). Abbreviations are in Figure 1. Box-and-whiskers plot with median, interquartile range and minimum and maximum values. *p<0.03 PSP-S vs. PSP-NS, C-NS and C-S. Images are representative of all samples.

Immunostaining with 4-HNE was used to assess lipid peroxidation in the lungs [15]. 4-HNE staining was present in all lung cells but mainly in macrophages. The staining was significantly higher for PSP-S than PSP-NS and C groups, for both increased staining intensity per macrophage and increased number of positive-stained macrophages (Figure 2 and Table 1, p<0.03).

**Table 1:** Quantification of HO-1-, BVR-, H-ferritin- and 4-HNE-positive macrophages (CD68 positive cells) in lung biopsies from C-NS, C-S, PSP-NS and PSP-S patients.

		C-NS	C-S	PSP-NS	PSP-S
**HO-1 staining**	0	15.2 [11.0–18.3]	14.1 [9.1–22.1]	13.2 [9.5–20.7]	2.7 [1.5–6.8] *
	+	78.9 [72.6–82.8]	80.3 [72.5–81.9]	76.3 [73.0–80.7]	16.2 [13.2–20.0] *
	++	6.9 [3.7–9.7]	5.4 [3.7–11.1]	9.4 [6.3–12.0]	79.7 [78.3–82.7] *
**BVR staining**	0	18.9 [15.5–22.7]	16.0 [13.7–23.2]	24.8 [17.0–33.3]	3.1 [1.8–8.0] *
	+	67.4 [56.4–73.2]	76.1 [67.2–79.2]	63.6 [59.2–72.7]	19.9 [17.2–25.1] *
	++	11.7 [9.7–26.4]	8.1 [6.3–10.0]	9.9 [7.5–13.7]	75.8 [71.6–78.8] *
**H-ferritin staining**	0	11.9 [9.5–15.6]	15.0 [9.6–25.0]	14.9 [10.5–30.1]	2.5 [0.6–6.7] *
	+	60.3 [57.5–72.9]	60.7 [54.8–64.2]	63.8 [50.6–71.2]	11.2 [3.0–18.9] *
	++	24.9 [17.7–32.8]	27.0 [12.7–28.3]	19.5 [16.8–24.3]	84.6 [77.6–96.4] *
**4-HNE staining**	0	24.0 [20.1–27.9]	17.0 [13.7–22.7]	20.0 [14.7–21.9]	0.5 [0.2–1.9] *
	+	73.8 [69.5–78.0]	79.4 [72.2–83.7]	77.7 [74.5–81.3]	6.7 [5.7–8.4] *
	++	2.4 [1.5–2.6]	3.8 [1.3–6.0]	3.2 [1.8–4.0]	92.6 [90.7–93.7] *

0  =  absent staining; +  =  moderate staining; ++  =  intense staining.

C-NS  =  control patient non-smokers; C-S  =  control patient smokers; PSP-NS  =  primary spontaneous pneumothorax non-smokers; PSP-S  =  primary spontaneous pneumothorax smokers.

HO-1  =  heme oxygenase-1; BVR  =  biliverdin reductase; 4-HNE  =  4-hydroxynonenal.

Results are presented as median [min-max] percentage, * p<0.03 vs C-NS, C-S, PSP-NS.

### Increased expression of HO-1 and other components of the HO system in PSP-S samples

**Figure 3:**
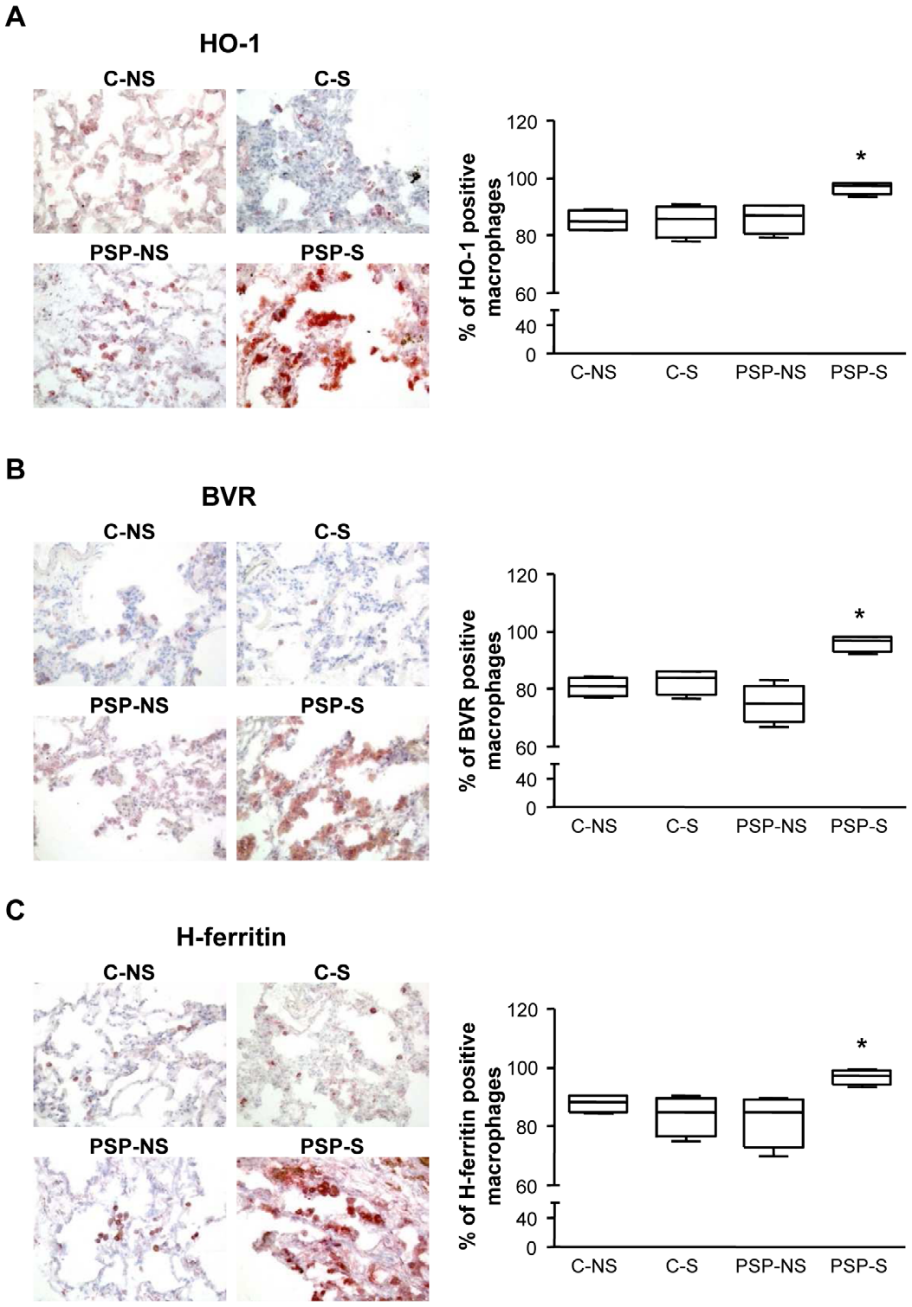
Detection of HO-1, BVR and H-ferritin in lung biopsies. Specific immunohistochemical staining and quantification of HO-1 (A), BVR (B), and H-ferritin (C) were assessed in lung biopsies from C-NS, C-S, PSP-NS and PSP-S patients (magnification ×200). Abbreviations are in Figure 1. Box-and-whiskers plot with median, interquartile range and minimum and maximum values. *p<0.03 PSP-S vs. PSP-NS, C-NS and C-S. Images are representative of all samples.

**Figure 4:**
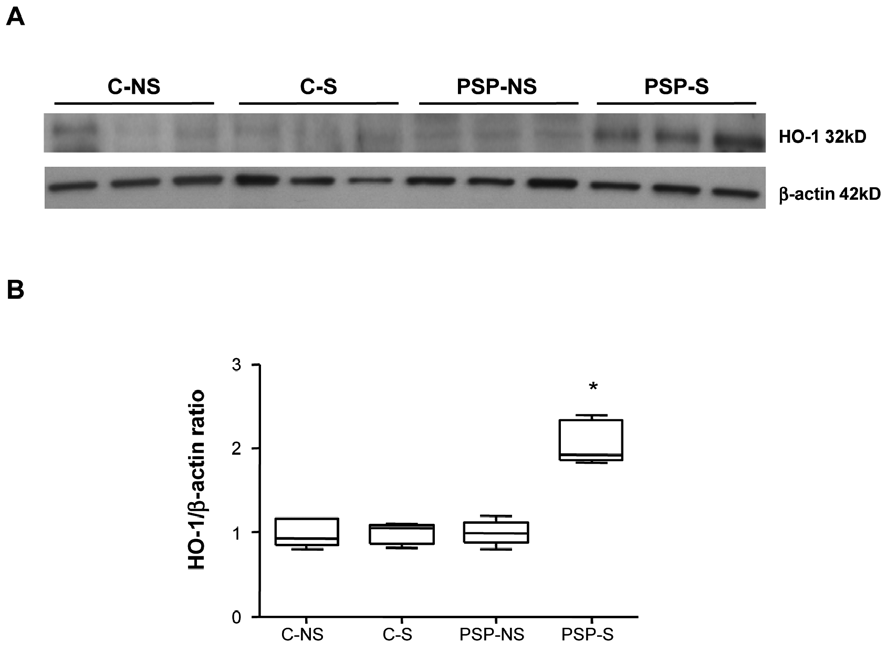
Expression of HO-1 protein in lung tissue. A: representative western blot of HO-1 protein expression of lung homogenates from C-NS, C-S, PSP-NS and PSP-S patients (n = 3 each) with the respective β-actin controls. Abbreviations are in Figure 1. B: quantification of HO-1 protein. Results are expressed as ratio of expression to that of β-actin (n = 5, * p<0.012 PSP-S vs. PSP-NS, C-NS and C-S).

Immunohistochemical staining for HO-1, BVR and H-ferritin in lung biopsies was significantly higher for PSP-S than PSP-NS and C patients (p<0.03; Figure 3), with both increased staining intensity per macrophage and increased number of positive-stained macrophages (Figure 3 and Table 1, p<0.03). Immunostaining with a CD68 antibody in sequential slides showed that most of the HO-1-positive cells in alveolar spaces were CD68 positive (88.5% [75.6–94.2]). The same result was observed with BVR (92.6% [82.7–98.2]) and H-ferritin (94.5% [85.6–98.5]). We further confirmed increased HO-1 expression at the protein level by western blot analysis of lung homogenates (Figure 4). Furthermore, induction of mRNA level of HO-1, BVR and H-ferritin in PSP-S lung biopsies was confirmed (Figure S1).

### Increased expression of HIF-1α but not Nrf2 in macrophages in PSP-S samples

**Figure 5:**
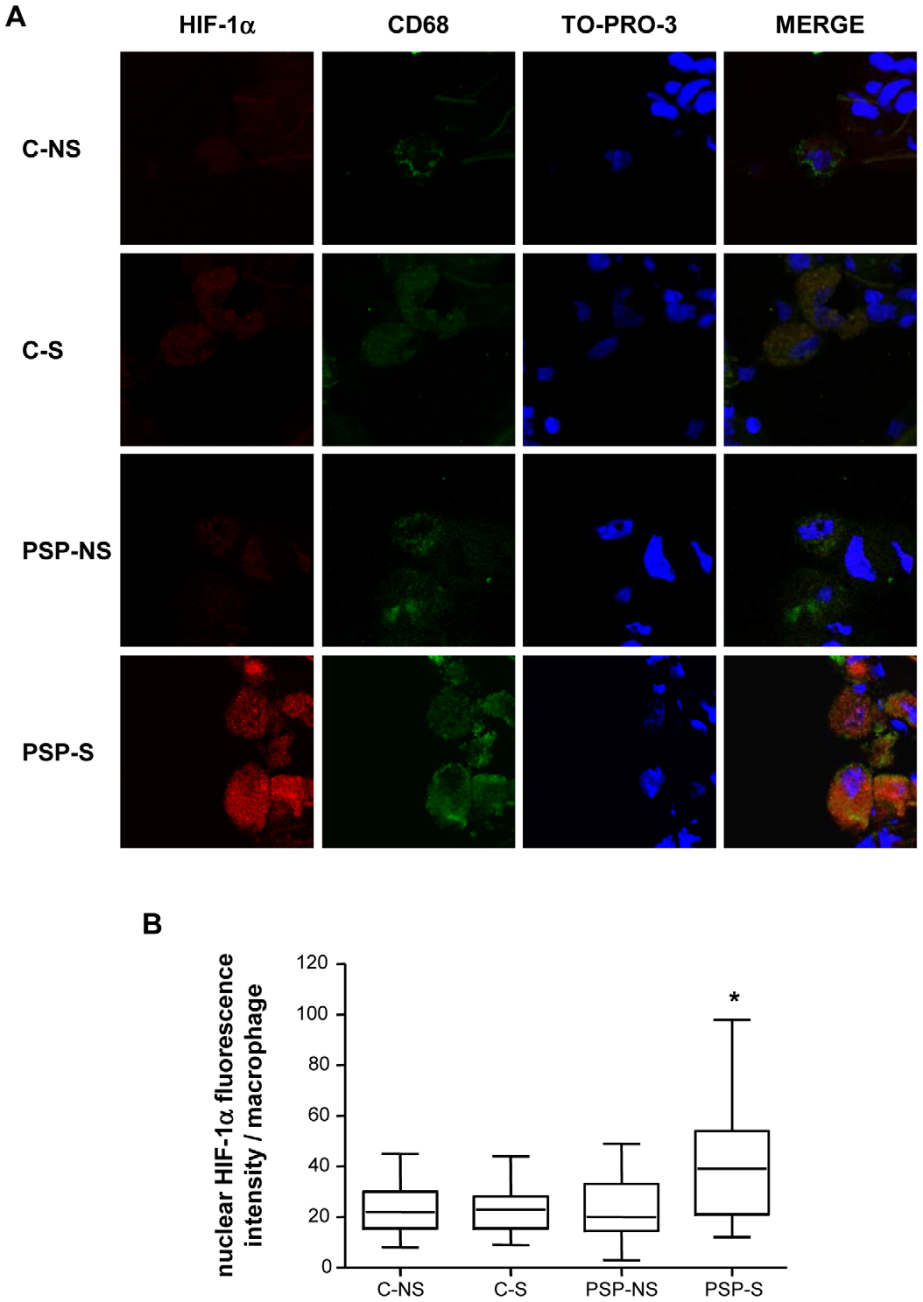
Confocal laser microscopy analysis of HIF-1α and CD68 expression in lung macrophages. A: Immunofluorescent staining was performed with HIF-1α (in red: left column) and CD68 (in green) and TO-PRO-3 DNA (blue). Co-expression is seen by double staining and overlays (Merge column) (magnification ×1200). B: Quantification of nuclear HIF-1α immunofluorescence in macrophages of C-NS, C-S, PSP-NS and PSP-S patients. Abbreviations are in Figure 1. Box-and-whiskers plot with median, interquartile range and minimum and maximum values. *p = 0.003 PSP-S vs. PSP-NS, C-NS and C-S. Images are representative of all samples.

**Figure 6:**
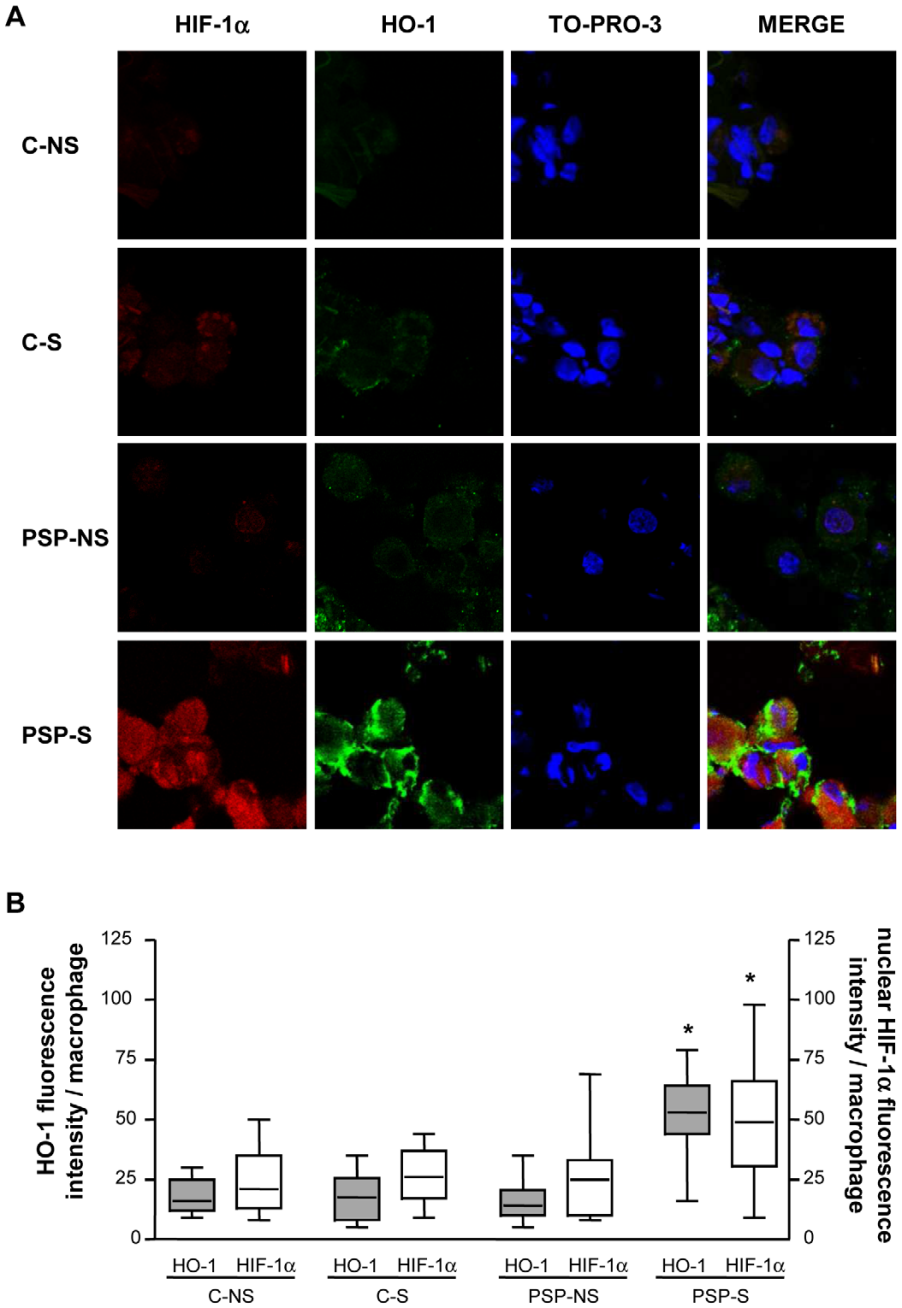
Confocal laser microscopy analysis of HIF-1α and HO-1 expression in lung macrophages. A: Immunofluorescent staining was performed with HIF-1α (in red: left column) and HO-1 (in green) and TO-PRO-3 DNA (blue). Co-expression is seen by double staining and overlays (Merge column). B: Quantification of nuclear HIF-1α immunofluorescence and cellular HO-1 immunofluorescence in macrophages of C-NS, C-S, PSP-NS and PSP-S patients (magnification ×1200). Abbreviations are in Figure 1. Box-and-whiskers plot with median, interquartile range and minimum and maximum values. *p<0.0001 for HO-1 and p = 0.001 for HIF-1α: PSP-S vs. PSP-NS, C-NS and C-S. Images are representative of all samples.

**Figure 7:**
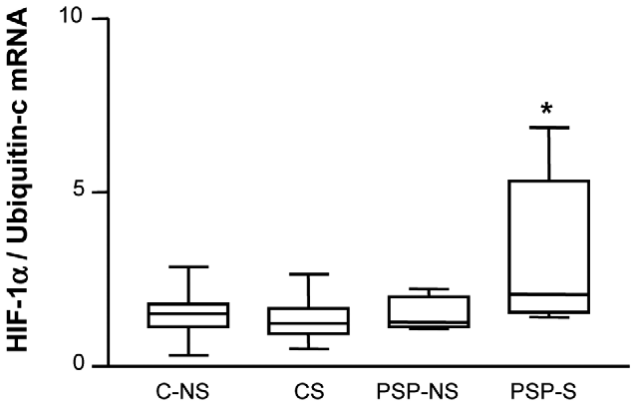
HIF-1α mRNA expression in lung tissue. Box-and-whiskers plot with median, interquartile range and minimum and maximum values. Abbreviations are in Figure 1. Results are expressed as ratio of expression to that of Ubiquitin-c. * PSP-S vs. C-NS: p = 0.03, vs. CS: p = 0.02, vs. PSP-NS: p = 0.04.

Because regulation of HO-1 transcription under oxidant exposure involves the transcription factors HIF-1α and Nrf2 and because macrophages are the main cells expressing HO-1, we investigated the nuclear expression of these transcription factors in macrophages in lung biopsies by confocal laser microscopy. The groups did not differ in nuclear expression of Nrf2 protein (p = 0.99; Figure S2). By contrast, HIF-1α protein expression in both the cytosol and nucleus of alveolar macrophages (CD68-positive cells) was higher in PSP-S tissue than in PSP-NS and C tissue, with no difference in expression between PSP-NS and C groups (Figure 5, p = 0.003 for the quantification of nuclear localization between PSP-S vs. the 2 other groups). This increased HIF-1α protein expression in macrophages was associated with increased HO-1 protein expression (Figure 6). The mRNA level of HIF-1α was also higher in PSP-S lung tissue than in PSP-NS or C tissue, which suggests transcriptional regulation (Figure 7).

### *In vitro* experiments

To further understand the regulation of HO-1, BVR and H-ferritin expression in alveolar macrophages, the THP-1 macrophage cell line was exposed to conditions mimicking the condition of C and PSP-S and PSP-NS patients: cigarette smoke condensate (CS) or air exposure followed or not by hypoxia and reoxygenation. The duration of exposure was as close as possible to the clinical setting: 24 h pre-exposure to CS, then 4 h hypoxia and up to 18 h reoxygenation (these 2 last time periods are similar to the time periods between symptoms and thoracostomy and between thoracostomy and thoracoscopic pleurodesis, respectively). The following nomenclature was adopted: R2, R4, R6 and R18, corresponding to 2, 4, 6 and 18 h, respectively, after the beginning of reoxygenation. We used 1 µg/ml CS because this dose did not induce HO-1 expression in THP-1 cells in normoxia. This condition mimicked the lack of induction of HO-1 expression observed in C-S patients.

### Effect of hypoxia/reoxygenation and CS exposure on HO-1 expression and related HO components

**Figure 8:**
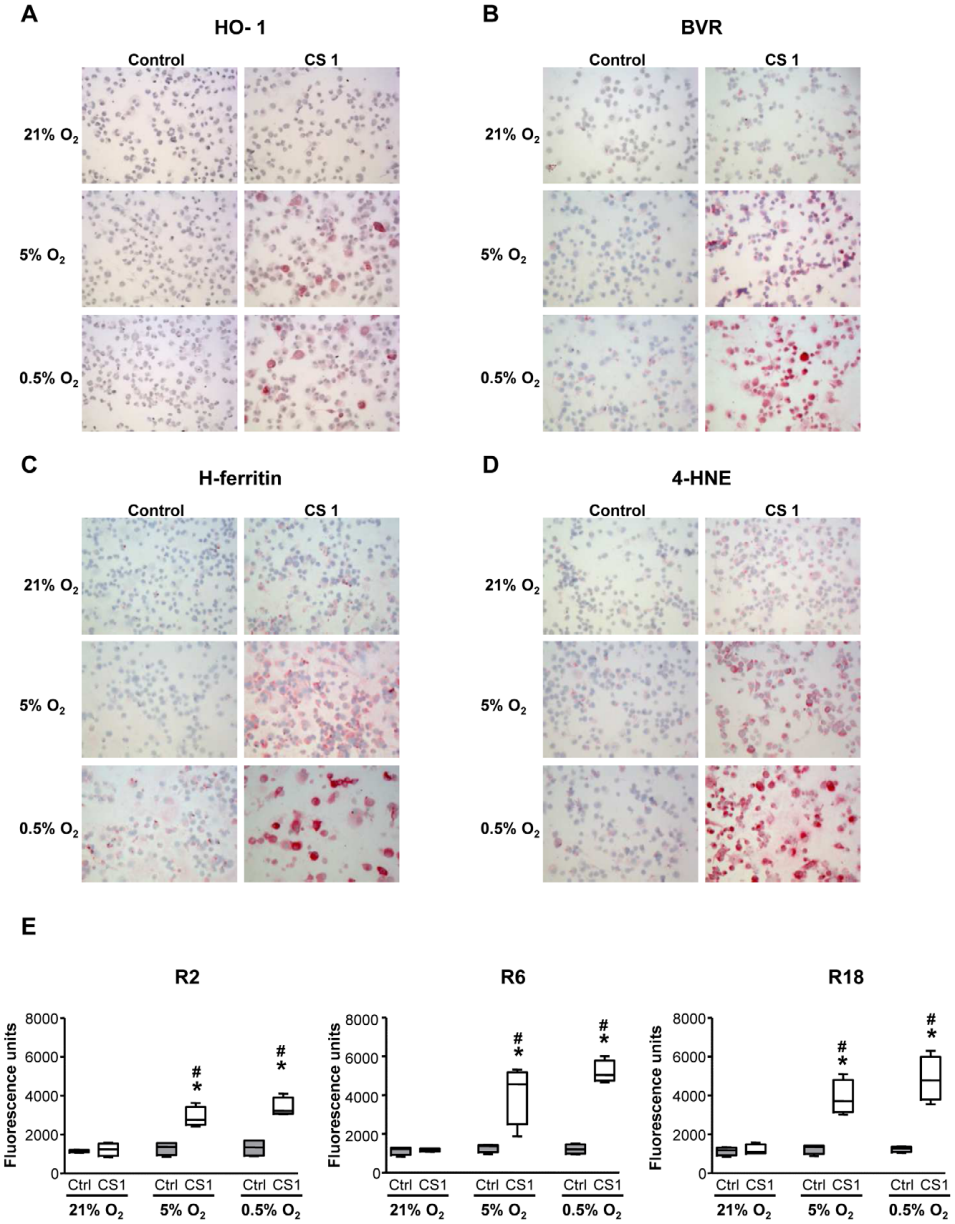
HO-1, BVR, H-ferritin, 4-HNE expression and ROS production in THP-1 cells. Specific immunocytochemical staining of HO-1 (A), BVR (B), H-ferritin (C) and 4-HNE (D) (magnification ×200) was performed in THP-1 cells exposed for 4 h to normoxic (21% O_2_) or hypoxic (5% and 0.5% O_2_) conditions and 1 µg/ml CS or DMSO (Control) 18 h after re-oxygenation (R18). No staining was observed when the specific antibodies were replaced by a control isotype antibody or a normal rabbit serum (data not shown). Images are representative of all samples. ROS production (E) was assessed by H_2_DCFH-DA oxidation 2 h (R2), 6 h (R6) and 18 h after re-oxygenation (R18) in 4 experiments in duplicate. Box-and-whiskers plot with median, interquartile range and minimum and maximum values. (n = 4, * p = 0.029 vs. Ctrl and # p = 0.029 vs. 21% O_2_).

Cells exposed to CS and hypoxia/reoxygenation did not differ from untreated or normoxic control cells in cell viability (data not shown). HO-1, BVR, and H-ferritin protein expression was not increased at the end of the whole experimental period (R18) in cells exposed to normoxia or hypoxia/reoxygenation without CS exposure (Figure 8A–C). By contrast, cells exposed to both CS and hypoxia/reoxygenation showed an increased expression of HO-1, BVR, and H-ferritin (Figure 8A–C). The level of expression of the components of the HO system was inversely related to O_2_ concentration, the main increase being observed in THP-1 cells exposed to 0.5% O_2_ (Table 2).

**Table 2:** Quantification of HO-1-, BVR-, H-ferritin- and 4-HNE-positive cells after hypoxia and 18 h reoxygenation for the indicated conditions.

		21% O_2_		5% O_2_		0.5% O_2_	
		Control	CS 1 µg/ml	Control	CS 1 µg/ml	Control	CS 1 µg/ml
HO-1 staining	0	100 [100–100]	99.7 [98.7–100]	100 [100–100]	1.1 [0–3.6] *, #	100 [100–100]	0 [0–3.8] *, #
	+	0 [0–0]	0.3 [0–1.3]	0 [0–0]	93.2 [88.5–95.5] * #	0 [0–0]	92.9 [90.9–95.4] * #
	++	0 [0–0]	0 [0–0]	0 [0–0]	5.7 [4.5–7.9] * #	0 [0–0]	5.3 [4.6–9.1] * #
BVR staining	0	93.5 [89.9–97.3]	91.4 [86.7–93.6]	91.2 [89.2–93.1]	11.3 [9.9–13.6] * #	84.5 [83.3–96.3]	0 [0–0] * # †
	+	6.5 [2.7–10.1]	8.6 [6.4–13.3]	8.8 [6.9–10.8]	84.1 [82.4–86.8] * #	15.5 [3.7–16.7]	1.8 [0–3] * # †
	++	0 [0–0]	0 [0–0]	0 [0–0]	4.3 [3.3–4.6] * #	0 [0–0]	98.2 [97–100] * # †
H-ferritin staining	0	97.9 [96.6–99.1]	89.4 [86.5–97.3]	97 [95.2–98.7]	3.6 [3.1–7.6] * #	88.7 [86.1–97.4]	0 [0–0] * # †
	+	2.1 [0.9–3.4]	10.6 [2.7–13.5]	3 [1.3–4.8]	91.8 [88.6–95.2] * #	11.3 [2.6–13.9]	10.1 [6.2–16.3] †
	++	0 [0–0]	0 [0–0]	0 [0–0]	3.2 [1–7.3] * #	0 [0–0]	89.9 [83.7–93.8] * # †
4-HNE staining	0	91.7 [87.8–95.9]	85.5 [71.4–93.1]	89.9 [83.8–94.3]	3.1 [1.2–5.4] * #	95.5 [93–98.6]	0 [0–0] * # †
	+	8.3 [4.1–12.2]	14.5 [6.9–28.6]	10.1 [5.7–16.2]	82.4 [78.8–88.9] * #	4.5 [1.4–7]	3.3 [1.4–6.5] # †
	++	0 [0–0]	0 [0–0]	0 [0–0]	14.3 [8.8–17.4] * #	0 [0–0]	96.7 [93.5–98.6] * # †

0  =  absent staining; +  =  moderate staining; ++  =  intense staining.

CS  =  cigarette smoke condensate; HO-1  =  heme oygenase-1; BVR  =  biliverdin reductase; 4-HNE  =  4-hydroxynonenal.

Results are presented as median [min-max] percentage, * p = 0.029 vs Control, **#** p = 0.026 vs 21% O_2_, † p = 0.029 vs 5% O_2_.

To better understand HO-1, BVR and H-ferritin induction mechanisms, we analyzed the kinetics of mRNA level changes during reoxygenation (Figure S3). CS exposure or hypoxia/reoxygenation without CS exposure did not induce HO-1 expression, whatever the duration of reoxygenation (Figure S3A). By contrast, moderate hypoxia (5% O_2_) potentiated CS-induced increase in HO-1 mRNA level. HO-1 mRNA expression was increased significantly at 4 h reoxygenation (R4) and was maintained until 6 h of reoxygenation (R6). This effect was more pronounced with severe hypoxia (0.5% O_2_). A similar time course effect was observed with BVR and H-ferritin mRNA levels, but the increased mRNA expression persisted until 18 h (Figure S3B-C).

### Effect of hypoxia/reoxygenation on oxidative stress

Staining for 4-HNE measured at 18 h reoxygenation (R18) was increased both in the cytosol and membrane of cells exposed to both CS and hypoxia/reoxygenation, with the main increase on exposure to 0.5% O_2_, which paralleled the increased HO-1, BVR and H-ferritin expression (Figure 8D and Table 2). Intracellular reactive oxygen species (ROS) production was significantly increased in cells exposed to both CS and hypoxia/reoxygenation as compared to CS alone or hypoxia/reoxygenation without CS exposure, thereby confirming results observed with 4-HNE staining (Figure 8E). This ROS production was observed from 2 h reoxygenation period (R2) to 18 h reoxygenation (R18).

### Effect of hypoxia/reoxygenation and CS exposure on HIF-1α and Nrf2 expression

To examine a possible cause-effect relationship between the increase in HIF-1á and HO-1, BVR and H-ferritin levels observed in PSP-S samples, we first investigated whether the increased expression of HO-1, BVR, and H-ferritin was associated with increased nuclear expression of HIF-1α in the different *in vitro* experimental conditions. In addition, we analyzed nuclear Nrf2 protein expression.

**Figure 9:**
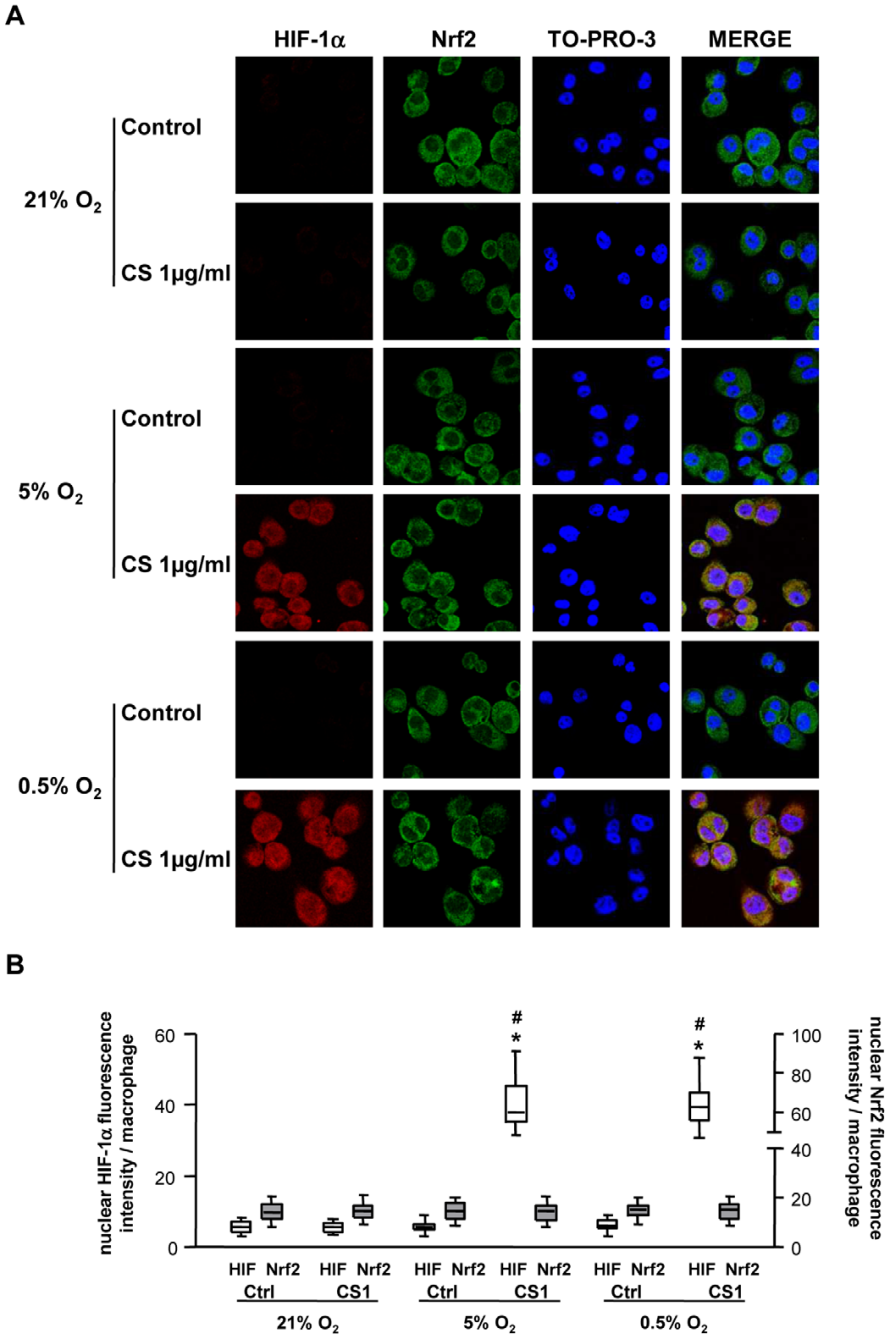
Expression of HIF-1α and Nrf2 protein in THP-1 cells at 4 h reoxygenation. HIF-1α and Nrf2 protein expression in THP-1 cells exposed to normoxic (21% O_2_) or hypoxic (5% and 0.5% O_2_) conditions and 1 µg/ml CS or DMSO (Ctrl) 4 h after reoxygenation (R4) was assessed by confocal laser microscopy analysis. A: Immunofluorescent staining involved HIF-1α (in red: left column), Nrf2 (in green) and TO-PRO-3 DNA (blue). Co-expression is seen by overlays (Merge column). B: Quantification of nuclear HIF-1α and Nrf2 immunofluorescence in THP-1 cells at R4. (magnification ×1200). Box-and-whiskers plot with median, interquartile range and minimum and maximum values. * p<0.0001 vs. Ctrl and # p<0.0001 vs. 21% O_2_.

**Figure 10:**
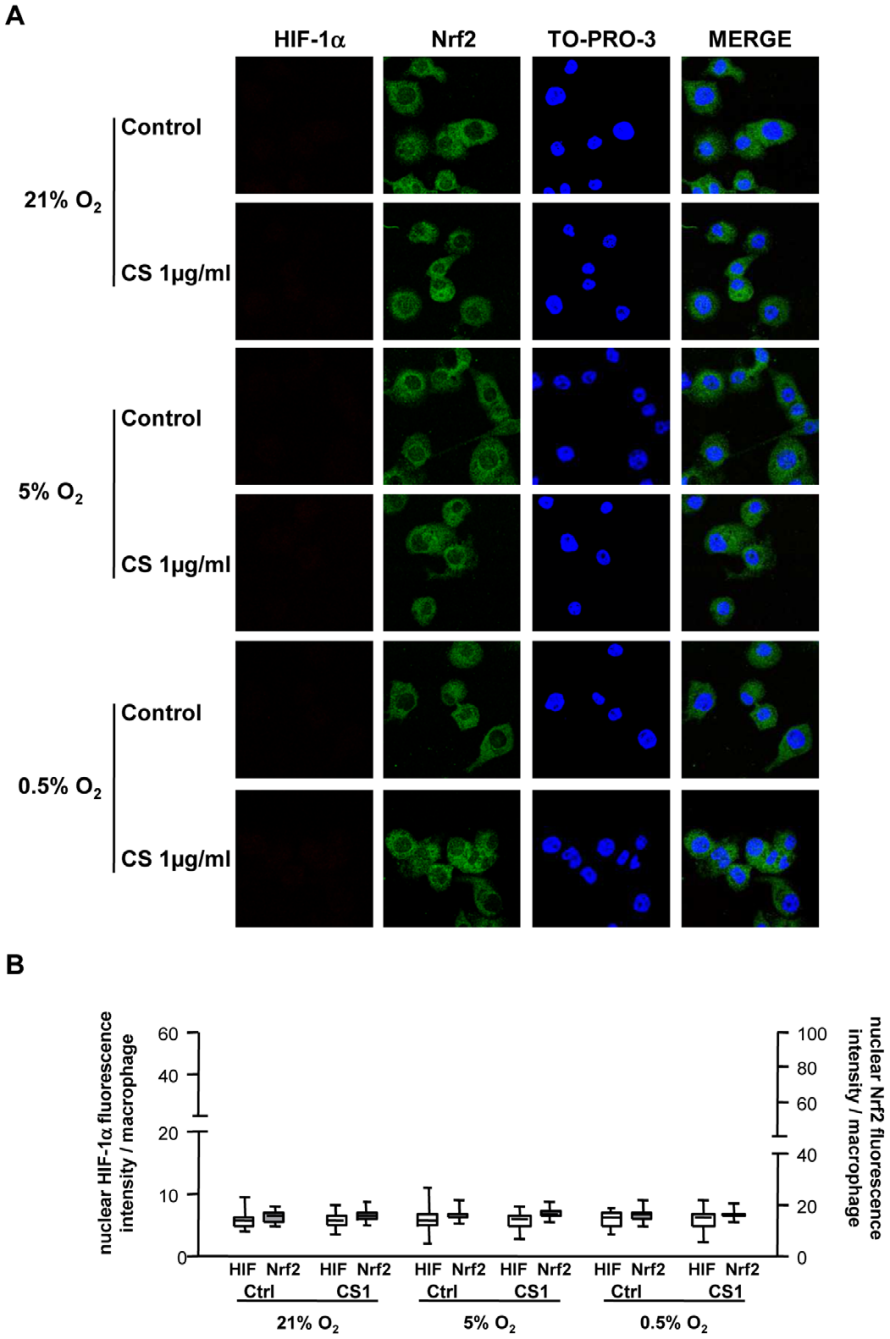
Expression of HIF-1α and Nrf2 protein in THP-1 cells at 6 h reoxygenation. HIF-1α and Nrf2 protein expression in THP-1 cells exposed to normoxic (21% O_2_) or hypoxic (5% and 0.5% O_2_) conditions and 1 µg/ml CS or DMSO (Ctrl) 6 h after reoxygenation (R6) was assessed by confocal laser microscopy analysis. A: Immunofluorescent staining involved HIF-1α (in red: left column), Nrf2 (in green) and TO-PRO-3 DNA (blue). Co-expression is seen by overlays (Merge column). B: Quantification of nuclear HIF-1α and Nrf2 immunofluorescence in THP-1 cells at R4. (magnification ×1200). Box-and-whiskers plot with median, interquartile range and minimum and maximum values.

Because the increase in HO-1, BVR and H-ferritin mRNA expression was observed at R4, we analyzed HIF-1α nuclear protein expression at this time point. HIF-1α nuclear protein expression was significantly increased in cells exposed to CS and hypoxia/reoxygenation at R4 and returned to the basal level at R6; Nrf2 nuclear protein expression showed no change (Figures 9–10). Cells exposed to CS or hypoxia/reoxygenation alone showed no modification of HIF-1α or Nrf2 expression (Figures 9–10). The increased HIF-1α protein expression in cells exposed to both CS and hypoxia/reoxygenation was confirmed at the mRNA level (Figure S4).

### Effects of HIF-1α silencing

**Figure 11:**
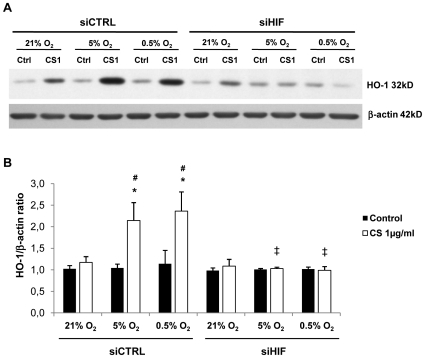
Effect of HIF-1α silencing on HO-1 protein expression in THP-1 cells. Cells were transfected with 300 nM siRNA for HIF-1α (siHIF) or nonsilencing siRNA (siCTRL). HO-1 protein expression was assessed by western blot analysis in transfected-THP-1 cells exposed to normoxic (21% O_2_) or hypoxic (5% and 0.5% O_2_) conditions and 1 µg/ml CS or DMSO (Ctrl) 18 h after reoxygenation (R18). A: Representative western blot of HO-1 protein expression in THP-1 cells 70 h after transfection with 300 nM siRNA for HIF-1α (siHIF) or nonsilencing siRNA (siCTRL), with β-actin control. B: Quantification of HO-1 protein. Results are expressed as a ratio to β-actin protein concentration (n = 4, *p =  0.005 vs. Ctrl, # p = 0.005 vs. 21% O_2_, ‡ p = 0.009 vs. siCTRL).

**Figure 12:**
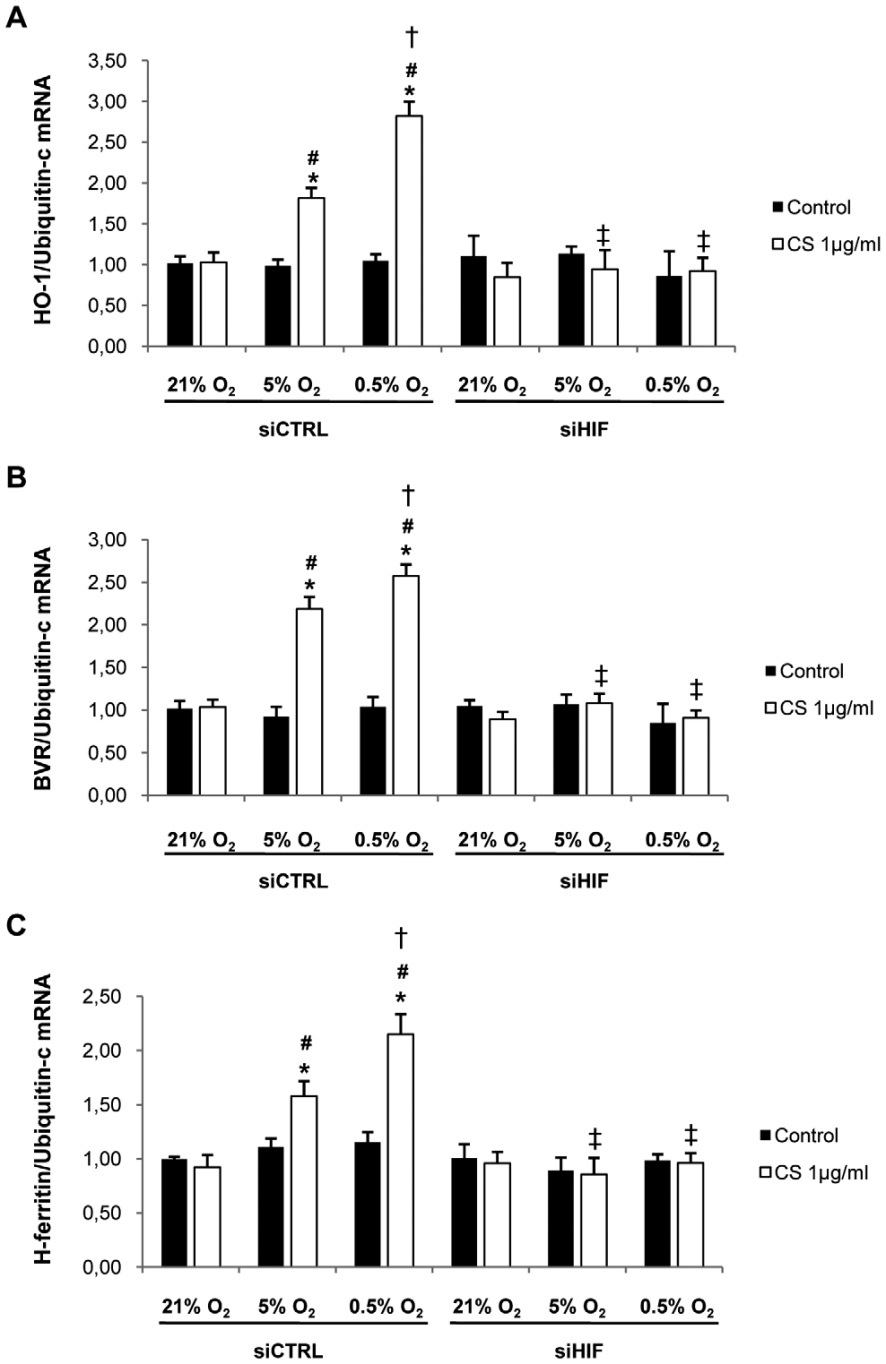
Effect of HIF-1α silencing on HO-1, BVR and H-ferritin mRNA expression in THP-1 cells. Cells were transfected with 300 nM siRNA for HIF-1α (siHIF) or nonsilencing siRNA (siCTRL). HO-1 (A), BVR (B) and H-ferritin (C) mRNA expression was assessed by quantitative RT-PCR analysis in transfected-THP-1 cells exposed to normoxic (21% O_2_) or hypoxic (5% and 0.5% O_2_) conditions and 1 µg/ml CS or DMSO (Ctrl) 4 h after reoxygenation (R4). Results are expressed as ratio of expression to that of Ubiquitin-c. (n = 4, *p =  0.029 vs. Ctrl, # p = 0.029 vs. 21% O_2_, † p = 0.029 vs. 5% O_2_, ‡ p = 0.029 vs. siCTRL).

To investigate the role of HIF-1α in HO-1, BVR and H-ferritin induction, we performed RNA silencing experiments. We first confirmed the efficiency of HIF-1α siRNA transfection by a decreased HIF-1α level both in cytosolic and nuclear compartments on confocal analysis and decreased HIF-1α mRNA expression (data not shown). Western blot analysis confirmed that HIF-1α siRNA transfection completely abrogated the increased HO-1 protein level in cells exposed to both CS and hypoxia/reoxygenation (Figure 11). HIF-1α siRNA transfection also completely abrogated the increased HO-1, BVR and H-ferritin mRNA levels, thus confirming the transcriptional regulation of HO-1, BVR and H-ferritin mRNA by HIF-1α (Figure 12).

## Discussion

This study shows that the expression in lung macrophages of both the oxidative stress marker 4-HNE and antioxidant and anti-inflammatory proteins HO-1, BVR and H-ferritin is significantly higher in patients with PSP exposed to cigarette smoke (PSP-S) than in patients with condition and not exposed (PSP-NS) and in control patients. The mRNA and protein expression and protein nuclear localisation of the transcription factor HIF-1α followed the same pattern as and colocalized with HO-1, whereas the expression and nuclear localisation of the transcription factor Nrf2 was not modified in patients with PSP. *In vitro* experiments in a human monocyte/macrophage cell line, THP-1, cultured under conditions mimicking the condition of lung macrophages during the pneumothorax condition and treatment in smokers (CS exposure with short hypoxia then reoxygenation) showed that HIF-1α was responsible for inducing HO-1, BVR and H-ferritin expression. Collectively, these results show that spontaneous pneumothorax in smokers is associated with lung macrophage oxidative stress and the orchestrated induction of the antioxidant proteins HO-1, BVR and H-ferritin, probably *via* an HIF-1α pathway.

Cigarette smoking is suggested as a risk factor for PSP [3] and is an independent risk factor for recurrence of the disease [4]. The present results shed some light in the mechanism(s) underlying this phenomenon because lung biopsies from PSP-S patients showed high macrophage infiltration and macrophages showed high lipid peroxidation (4-HNE staining) as compared with lung biopsies from PSP-NS patients. Our results agree with data published previously showing inflammatory changes in lung samples from patients with PSP [5], [16]. However, in previous studies, the effect of CS exposure was only slightly investigated. Furthermore, no analysis of oxidative stress was performed.

Oxidative stress can predispose to the development and recurrence of PSP by amplifying and/or perpetuating inflammation [1], [17], which thus provides new elements to understand the pathophysiology of this disease. The difference between PSP-S and PSP-NS patients could be related to time between symptoms and thoracostomy; some studies have found an association of duration of air in the pleural space and the inflammatory response in the pleural fluid [18]. However, this situation did not occur in our study because the time between symptoms and thoracostomy and between thoracostomy and surgical pleurodesis was similar for all patients. Moreover, the number and size of previous pneumothoraces were similar for PSP-S and PSP-NS patients according to patient history. A prospective study with a detailed description of events during pneumothorax condition and treatment would be more relevant to assess differences in oxidative stress between PSP-S and PSP-NS patients.

To counteract oxidative stress, the organism possesses several systems. In PSP-S patients, we found a highly orchestrated response involving the induction of HO-1, BVR and H-ferritin expression. Although the induction of HO-1 expression has been demonstrated in several respiratory pathological conditions such as emphysema, hyperoxia [19], influenza virus infection [20], ozone-induced lung injury [21] and asthma [22], its involvement in PSP has not been shown before. Furthermore, no study has demonstrated the concomitant induction of BVR and H-ferritin, along with HO-1, in pulmonary and nonpulmonary pathological conditions. This coordinated response is important from a functional point of view. Indeed, recent studies showed that BVR and H-ferritin play critical roles in mediating the cytoprotective effects of HO-1 against hypoxia/reoxygenation injury in cardiomyocytes, hepatocytes and endothelial cells [23], [24]. Of note, no induction of HO-1 expression in control smokers was unexpected. However, these results agree with previous results from our laboratory and other groups [9], [25], [26]. Moreover, no staining for 4-HNE in these patients reflects an absence of oxidative stress, which thus supports no induced expression of the redox-sensitive HO-1. PSP patients were younger than control patients and had less cumulative tobacco exposure. These differences in dose and duration of cigarette exposure could influence the qualitative and quantitative induction of the antioxidant HO-1 system in macrophages, because a dose effect and biphasic expression were evidenced *in vitro* [10].

During the pneumothorax condition and treatment, atelectasis and reexpansion are associated with acute alveolar hypoxia and reoxygenation, respectively [12]. We found induced expression of HIF-1α, a transcription factor involved in hypoxia signaling events, and it co-localized with HO-1 in macrophages from PSP-S patients. This finding suggests a role of HIF-1α in inducing HO-1 expression in this condition. Since hypoxia/reoxygenation alone without CS exposure did not induce HIF-1α in macrophages of PSP-NS patients, our data suggest that CS preconditioning is required for the hypoxia/reoxygenation induction of HIF-1α expression. Such a relationship was further supported by *in vitro* experiments in macrophages exposed to CS then hypoxia and reoxygenation, to mimic conditions occurring during the pneumothorax condition and treatment in smokers [12]. In these experiments, HO-1 induction was suppressed by selective HIF-1α gene silencing. This finding agrees with studies showing a role of HIF-1α in inducing HO-1 expression in different cell types [27], [28]. However, recently, Li et al. suggested that hypoxia and ethyl-3,4-dihydroxybenzoate (a prolyl-hydroxylase inhibitor) treatments differentially regulate HO-1 expression through HIF-1α-independent pathways and that HIF-1α is unlikely to function as a key regulator for HO-1 in human cell lines [29]. These findings fit well with our data. Indeed, with no CS exposure, as in PSP-NS patients and THP-1 cells exposed only to hypoxia, the expression of HIF-1α and, subsequently HO-1 was not induced. However, the moderate duration and/or intensity of the hypoxia period could explain this finding. For example, Knowles et al. [30] reported HIF-1α protein accumulation in PMA-differentiated THP-1 cells after 16 h of 0.1% O_2_ hypoxia, values that are greater than our conditions (4 h at 5% and 0.5% O_2_). Similar protocols were used in alveolar epithelial cells to show induced HIF-1α expression by hypoxia [31]. Interestingly, HIF-1α gene silencing abrogated the induced expression of not only HO-1 but also BVR and H-ferritin. We believe this is the first demonstration of a role of HIF-1α in inducing BVR and H-ferritin gene expression. These data are in line with recent results showing that hypoxia induces BVR expression in the human proximal tubule cell line HK-2 [32]. Moreover, in previous studies, HIF-1α was shown to transcriptionally regulate the expression of proteins such as transferrin involved in iron homeostasis [33].

Because HIF-1α was upregulated only in PSP-S patients and in hypoxia/reoxygenation cells, but only when they were pre- and concomitantly exposed to CS, we investigated the mechanism(s) underlying this facilitator effect of CS. We focused on Nrf2 because this transcription factor is a central player in inducing the expression of antioxidant genes in response to a xenobiotic aggression, such as CS exposure [34], [35]. However, Nrf2 protein expression was not induced in C-S or PSP-S patients or in cells exposed to CS and hypoxia/reoxygenation. Nrf2 seems not to play a major role in HO-1 induction in PSP-S patients.

This study has some limitations. First, the number of patients in each group was low, especially in the PSP-NS group. This limited number of patients is justified by our intention to include patients with a similar time course between symptoms and thoracostomy and between thoracostomy and surgical pleurodesis, to facilitate the comparison between groups. Second, the association of data from patient lung tissue and *in vitro* data may be questioned. A major aim of the study was to provide a mechanistic explanation for the results observed in patients. Therefore, we chose the cell type in which the expression of HO-1 and related proteins was induced (macrophages) and designed an experimental set-up to mimic as close as possible conditions occurring during the pneumothorax condition and treatment in smokers. Although this strategy has limitations, the concordance of most of the results concerning HO-1 and related protein expression and HIF-1α expression between data from patients and *in vitro* data supports the validity of this approach. Moreover, THP-1 cells exposed to CS allowed us to mimic and provide mechanisms potentially involved in decreased HO-1 expression in alveolar macrophages in smoking-induced pulmonary emphysema [9], [10]. Our results do not explore the link between redox changes and PSP pathophysiology (i.e., our results do not explain how subpleural blebs occur and promote PSP development). Because our results were observed in PSP-S and not PSP-NS patients argues against a phenomenon of pure re-expansion acute lung injury but deserves further investigation. A mouse model of provoked pneumothorax would be helpful but does not mimick spontaneous pneumothorax. Therefore, candidate transcription factors involved in the link between oxidative stress and hypoxia/reoxygenation in macrophages in this model may be non relevant in human.

In conclusion, although the primary mechanisms responsible for spontaneous pneumothorax remain to be elucidated, the present study suggests that the pathophysiology of the disease could differ between smokers and nonsmokers and provides a new link between oxidative stress and hypoxia/reoxygenation in macrophages. Furthermore, this study may contribute to a better knowledge of the pathophysiology of other conditions associated with cigarette smoke exposure in the lung and hypoxia/reoxygenation, such as consequences of ischemia reperfusion with lung surgery or vascular diseases.

## Materials and Methods

### Patients

This study was approved by the local ethics committee, and stored biopsies were reported to our institutional board (Delegation a la Recherche Clinique, Assistance Publique - Hopitaux de Paris, Carre Historique - Hopital Saint-Louis, 1 avenue Claude Vellefaux, 75475 Paris Cedex 10, France/Comite de Protection des Personnes, Ile de France 1, 1 Place du Parvis Notre-Dame, 75181 Paris cedex 04, France, reference 0811760). Written consents were not collected since our biological collection was constituted before 2004 and confirmed as surgical waste with the approval of our institutional review board. However, all patients, informed orally, agreed that part of their biopsies could be used for research, and that the data of our biological collection were analyzed anonymously with the approval of our institutional review board. All patients included in this study gave their verbal consent, which was orally recorded by medical staff, prior to surgery.

#### Patients with primary spontaneous pneumothorax

Fifteen patients undergoing surgical pleurodesis and diagnostic lung biopsies for recurrent primary spontaneous pneumothorax (PSP) were included. Nine patients were smokers (PSP-S group) and 6 were nonsmokers (PSP-NS group). Tissue samples were taken from the resected parenchyma adjacent to pleura. PSP-S and PSP-NS patients showed similar number and size of previous occurrence of pneumothoraces, as well as time between symptoms and chest tube thoracostomy (4–8 h) and between thoracostomy and biopsies (24–48 h).

#### Control patients

Normal tissue was obtained from 19 control patients (C). Nine patients were smokers (control smoker group, C-S) and 10 were nonsmokers (control nonsmoker group, C-NS). C-S patients were undergoing surgery for resection of a localised primary lung carcinoma (n = 8) or a benign lesion (n = 1). C-NS patients were undergoing surgery for resection of a localised primary lung carcinoma (n = 6), lung metastases (n = 1) or a benign lesion (n = 3). Tissue samples were taken at a site distant from the pathological process, in macroscopically and microscopically normal regions adjacent to pleura.

Clinical characteristics of patients are in Table S1. As expected, patients with PSP were younger than C patients, and their cumulative tobacco exposure was lower than that of C-S patients.

### Processing of lung samples

Lung tissue fragments (about 0.2 cm^3^) were immediately frozen in liquid nitrogen and stored at −80°C for RNA, protein and immunostaining analysis. The histopathologic features of biopsies were evaluated on paraffin-embedded sections to exclude features of emphysema, necrotizing infections, neoplasms, Langerhans' cell histocytosis, lymphangioleiomyomatosis and pulmonary fibrosis.

### Quantitative RT-PCR analysis

Total RNA was extracted from frozen lung tissue and THP-1 cells (Nucleospin RNAII, Macherey Nagel, Düren, Germany) and reverse transcribed. HO-1, BVR, H-ferritin, HIF-1α mRNA expression was quantified by quantitative real-time PCR (MX3000P, Stratagene, La Jolla, CA) as described [36] and expressed as a ratio of expression to that of Ubiquitin-c (Table 3).

**Table 3:** Sequence of primer pairs used for amplification of cDNAs.

Gene	Forward primer (5′ to 3′)	Reverse primer (5′ to 3′)
HO-1	TTCTTCACCTTCCCCAACATTG	CAGCTCCTGCAACTCCTCAAA
BVR	CCACTTTGGAAGAGCGAAAGG	GACCCAGACTTGAAATGGAAGCT
H-ferritin	CACAAACTGGCCACTGACAAAA	CCCAATTCTTTGATGGCTTTCA
HIF-1α	CATTACCCACCGCTGAAAcG	CTTGATTGAGTGCAGGGTCAGC
Ubiquitin-c	CACTTGGTCCTGCGCTTGA	TTTTTTGGGAATGCAACAACTTT

HO-1  =  heme oygenase-1; BVR  =  biliverdin reductase; HIF-1α  =  hypoxia inductible factor-1α.

### Immunohistochemical analysis

Immunohistochemistry was performed as previously described [9]. Cryostat sections 4-6 µm thick or chamber slides (THP-1 cells) were fixed in acetone and reacted with appropriate dilutions of antibodies. Primary antibodies were against human HO-1 (1∶1000 dilution, Stressgen, Le-Perray-en-Yvelines, France), BVR (1∶1000, Stressgen), CD68 (macrophage marker; 1∶50 Clone PG-M1; Dako, Glostrup, Denamark), Major Basic Protein (MBP; eosinophil marker; 1∶30, Clone BMK13; Oxford Biotechnology Ltd, Kidlington, UK), H-ferritin (1∶10000 rHO_2_ kindly provided by Dr Paolo Santambrogio, San Raffaele Institute, University of Milano, Italy), elastase (neutrophil marker; 1∶100, Clone NP57, Dako) and 4-HNE (1∶5000, Calbiochem, San Diego, CA).

Positive cells were revealed by use of the Vectastain ABC-alkaline phosphatase kit (Vector Laboratories, Burlingame, CA) and the fast red substrate (Dako AAPAAP kit system, Glostrup, Denamark). To test the specificity of the immunostaining, antibodies were replaced by an isotype-matched control antibody or normal rabbit serum, with no positive cells identified (data not shown). On adjacent sections, positive cells with characteristic macrophage morphology were evaluated in 10 different high-power fields at ×200 magnification by 2 independent observers, and results were expressed as the number of positive cells per millimeter square lung tissue for CD68, MBP and elastase or as percentage of CD68-positive macrophages positive for HO-1, BVR, H-ferritin or 4-HNE. The intensity of protein staining in lung biopsies and THP-1 cells was graded from 0 (absent) to ++ (intense staining). Complete agreement in scoring was obtained between the 2 independent observers.

### Confocal laser scanning microscopy

Double-immunofluorescence labeling was performed to colocalize CD68 or HO-1 with HIF-1α or Nrf2 in lung biopsies and Nrf2 with HIF-1α in THP-1 cells, as previously described [9]. Cryostat sections 10 µm thick and THP-1 cells in chamber slides were fixed in 4% paraformaldehyde, saturated with 50 mM NH_4_Cl and permeabilised with 0.1% Triton X100. After saturation with normal serum from the species used to produce the secondary antibodies, slides were incubated with the appropriate dilution of primary antibodies (1∶10 HIF-1α [Santa Cruz Biotechniology, Santa Cruz, CA], 1∶25 Nrf2 [Santa Cruz Biotechnology], 1∶10 CD68; 1∶1000 HO-1) before the addition of secondary fluorescent-labelled antibodies (1∶750 dilution) (for HIF-1α, Alexa Fluor 568-conjugated antimouse; for Nrf2, Alexa Fluor 488-conjugated antirabbit for THP-1 cells and Alexa Fluor 546-conjugated antirabbit for lung biopsies; for CD68 and HO-1, Alexa Fluor 488-conjugated antimouse and antirabbit, repectively) and treated with RNAse A (1 mg/ml). Cell nuclei were localized on DNA staining with TO-PRO-3 (0.4 µg/ml) (Molecular Probes, Oregon, USA). Slides were mounted with Prolong Gold anti-fade reagent (Invitrogen, Carlsbad, CA) and examined by confocal laser scanning microscopy (LSM-510-META microscope; Zeiss, Oberkochen, Germany). To quantify fluorescence intensity per macrophage, macrophage area was delimited for 100 macrophages. For cellular fluorescence intensity, the total number of pixels corresponding to Alexa Fluor 568 or Alexa Fluor 488 fluorescence in these areas was determined. For nuclear fluorescence intensity, the total number pixels corresponding to Alexa Fluor 568 or Alexa Fluor 488 fluorescence associated with TO-PRO-3 (nucleus marker) fluorescence was subsequently determined (Start LSM Image browser). Results were expressed as the intensity of cellular HO-1 immunofluorescence or nuclear HIF-1α and Nrf2 immunofluorescence per macrophage. No labelling was observed when primary antibodies were replaced by appropriate isotype-matched control antibodies or normal serum.

### Preparation of lung homogenates for western blot analysis

Lung biopsy samples were homogenised in 10 mM Tris-HCl, pH 6.8, 1% SDS, 5% glycerol. Aliquots were stored at −80°C with 10% Protease Inhibitor Cocktail (Sigma-Aldrich, Saint Quentin Fallavier, France). Western blot analysis was performed as described [9] with anti-HO-1 antibody (1∶3000). Results are expressed as a ratio of expression to that of β-actin (1∶3000; monoclonal anti-β-actin antibody, Sigma-Aldrich).

### *In vitro* experiments

#### Cell culture and cigarette smoke condensate

The THP-1 cell line was purchased from the American Type Culture Collection (TIB202, ATCC, Rockville, MD). Cells were grown as previously described [10] in RPMI1640 in the presence of 10% fetal calf serum (FCS), 5.10^−5^ M β-mercapto-ethanol, 4 mM L-glutamine, 100 U/ml penicillin and 100 µg/ml streptomycin (complete medium) and differentiated with PMA (30 ng/ml) for 24 h. Cells were washed and re-incubated in complete medium with 0.1-1-10 µg/ml cigarette smoke condensate (CS) dispersed in 0.1% dimethyl sulfoxide (DMSO) in preliminary experiments, as previously described [10]. CS concentration was expressed as micrograms per milliliter for comparison with literature data [37].

CS was prepared by the use of a smoking machine (Anitech, Paris, France). The particulate matter from Kentucky standard cigarettes (2R4F; University of Kentucky, KY, USA) was collected on Cambridge glass fiber filters, and the amount obtained was determined by weight increase of the filter. The collected smoke particulates were dissolved in DMSO to yield a 20 mg/ml solution, and aliquots were stored at −80°C.

The control condition consisted of cells incubated in DMSO at the same concentration as for cells cultured in the presence of CS.

#### Exposure to hypoxia and reoxygenation

For both normoxia and hypoxia exposure, cells were overlayed with a thin layer of complete medium (0.2 ml/cm^2^) to decrease the diffusion distance of the ambient gas. Cells were placed in a humidified 12 L airtight Lwoff chamber (Lequeux, Paris, France) as previously described [38] and flushed with the following gas mixtures (Air Liquid, Paris, France): 5% CO_2_/90% N_2_/5% O_2_ (moderate hypoxia), 5% CO_2_/94.5% N_2_/0.5%O_2_ (severe hypoxia) and 5% CO_2_/74% N_2_/21% O_2_ (normoxia control). The gas was infused continuously into the chamber at 5 L/min for 10 min through a manometer (Air Liquid, HBS 300/1, Paris, France) placed at the entrance of the chamber as previously described under oxy-capnometer control (Datex Ohmeda, Trappes, France) [38]. Cells were exposed to these conditions at 37°C for 4 h and then transferred to the normoxic condition for 2-, 4-, 6- and 18-h reoxygenation. The PO_2_ levels measured in culture medium at the steady state were 5.3, 9.3 and 18.7 kPa for 0.5%, 5%, and 21% O_2_, respectively (ABL800, Radiometer Medical, Brønshøj, Denmark). Normoxia and hypoxia conditions did not differ in pH of culture medium.

#### Cellular toxicity

Cellular toxicity and viability were assessed by lactate dehydrogenase (LDH) release in the medium (cytotoxicity detection kit LDH, Roche, Meylan, France) and trypan blue exclusion test [10].

#### Intracellular ROS production

Intracellular ROS production was quantified by measuring 2′,7′-dichlorodihydrofluorescein diacetate (H_2_DCFH-DA) oxidation as previously described [39]. Briefly, H_2_DCFH-DA (final concentration in DMSO, 10 µM) was added 1 h before THP-1 cells stimulation in 96-well plates. Fluorescence was measured at 480–555 nm with use of a multiwell fluorescence plate reader (Fluorostar BMG). Results were expressed in fluorescent arbitrary units.

#### Preparation of THP-1 protein extracts for western blot analysis

Whole cell extracts were obtained by lysing THP-1 cells in lysis buffer (150 mM NaCl, 10 mM HEPES, pH 8, 500 mM saccharose, 1 mM Na_2_EDTA, 1% Nonidet P40). Aliquots of cellular extracts were stored at −80°C with 10% protease inhibitor cocktail. Protein concentration was determined by the Bradford method (Quickstart, Bio-Rad, Marnes-la-Coquette, France). Western blot analysis was performed as described [10]. Primary anti-HO-1 was the same as described previously (1∶3000 dilution). Protein expression was quantified by densitometric analysis under light with use of a charge-coupled device camera and an image analyzer (VisioCapt-Bio1D, Fisher Bioblock Scientific, Illrisch, France). Results are expressed as ratio of expression to that of β-actin (1∶3000 dilution).

#### siRNA transfection

Cells were transfected with 300 nM HIF-1α small interfering RNA (siRNA; NM_181054 Dharmacon SMARTpool siRNA reagent; Dharmacon, Lafayette, CO) or negative control siRNA (Dharmacon plus nontargeting pool) with Transpass R2 transfecting reagent (New England BioLabs, Ipswich, MA) as per the manufacturers' instructions [9].

Briefly, HIF-1α or negative control siRNA was added to the transfecting reagent diluted in serum-free medium and incubated for 20 min for the formation of the transfection complex. The siRNA transfection complexes were added at a final concentration of 300 nM to 5×10^5^ cells/well in 6-well plates (for western blot analysis) or 10^5^ cells/well in chamber slides (for confocal analysis) and incubated for 4 h, then fresh complete medium was added. Target protein knockdown was assessed 24 to 70 h posttransfection by confocal analysis with anti- HIF-1α antibody and RT-PCR. Under these conditions, the transfected cells looked morphologically normal, and viability did not differ from that for untransfected cells.

### Statistical analysis

Data were analysed by use of Statview software (Abacus Concepts, Inc.) and displayed for patients as medians and ranges and for *in vitro* data as mean and SD. Between-group differences were first assessed by nonparametric ANOVA (Kruskal-Wallis test). In the case of global significant difference, between-group comparisons were assessed by nonparametric Mann-Whitney U-test. Categorical data were analysed by chi-square test. A *p*<0.05 was considered statistically significant.

## Supporting Information

Figure S1 Expression of HO-1 (A), BVR (B) and H-ferritin (C) mRNA in lung tissue from C-NS, C-S, PSP-NS and PSP-S patients. C-NS and C-S, control patient nonsmokers and smokers, respectively; PSP-NS and PSP-S, primary spontaneous pneumothorax nonsmokers and smokers, respectively. Box-and-whiskers plot with median, interquartile range and minimum and maximum values. Results are expressed as ratio of expression to that of Ubiquitin-c. * for HO-1: PSP-S vs. C-NS: p = 0.0004, vs. CS: 0.0003, vs. PSP-NS: p = 0.02; for BVR: PSP-S vs. C-NS: p = 0.004, vs. CS: p = 0.002, vs. PSP-NS: p = 0.02; for H-ferritin: PSP-S vs. C-NS: p = 0.002, vs. CS: p = 0.004, vs. PSP-NS: p = 0.003.
(0.21 MB TIF)

Figure S2 Confocal laser microscopy analysis of Nrf2 expression in lung biopsies. Quantification of nuclear Nrf2 immunofluorescence in macrophages of C-NS, C-S, PSP-NS and PSP-S patients. Abbreviations are in Figure S1. Box-and-whiskers plot with median, interquartile range and minimum and maximum values. (p = 0.99).
(0.09 MB TIF)

Figure S3 mRNA expression of HO-1 (A), BVR (B) and H-ferritin (C) in THP-1 cells exposed to normoxic (21% O2) or hypoxic (5% and 0.5% O2) conditions and 1 µg/ml CS or DMSO (Control) 4 h (R4), 6 h (R6) and 18 h (R18) after reoxygenation. *p =  0.029 vs. Ctrl, # p = 0.029 vs. 21% O2, + p = 0.029 vs. 5% O2.
(0.66 MB TIF)

Figure S4 HIF-1alpha mRNA expression in THP-1 cells exposed to normoxic (21% O2) or hypoxic (5% and 0.5% O2) conditions and 1 µg/ml CS or DMSO (Control). Panel A: at 2 h reoxygenation (R2), panel B: at 6 h reoxygenation (R6), panel C: at 18 h reoxygenation (R18). Results are expressed as ratio of expression to that of Ubiquitin-c. *p =  0.029 vs. Ctrl and # p = 0.029 vs. 21% O2.
(0.30 MB TIF)

Table S1 Clinical characteristics of patients.
(0.03 MB DOC)
