# Supplementary figures and images for: Induction of Heme Oxygenase-1, Biliverdin Reductase and H-Ferritin in Lung Macrophage in Smokers with Primary Spontaneous Pneumothorax: Role of HIF-1α
Source: PLoS One. 2010 May 28;5(5):e10886. doi: 10.1371/journal.pone.0010886 (PMC2878337; doi:10.1371/journal.pone.0010886)

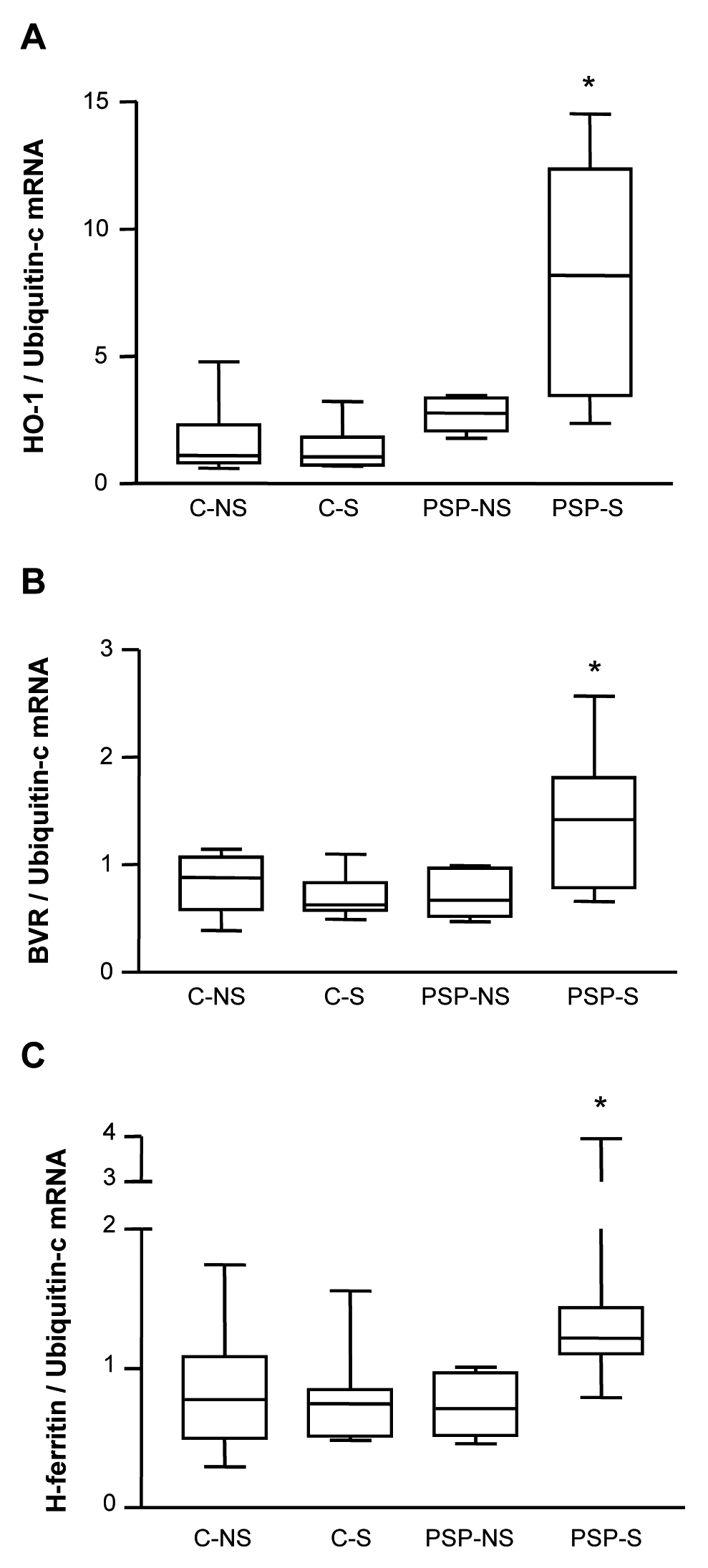

Supplement: Figure S1 — Expression of HO-1 (A), BVR (B) and H-ferritin (C) mRNA in lung tissue from C-NS, C-S, PSP-NS and PSP-S patients. C-NS and C-S, control patient nonsmokers and smokers, respectively; PSP-NS and PSP-S, primary spontaneous pneumothorax nonsmokers and smokers, respectively. Box-and-whiskers plot with median, interquartile range and minimum and maximum values. Results are expressed as ratio of expression to that of Ubiquitin-c. * for HO-1: PSP-S vs. C-NS: p = 0.0004, vs. CS: 0.0003, vs. PSP-NS: p = 0.02; for BVR: PSP-S vs. C-NS: p = 0.004, vs. CS: p = 0.002, vs. PSP-NS: p = 0.02; for H-ferritin: PSP-S vs. C-NS: p = 0.002, vs. CS: p = 0.004, vs. PSP-NS: p = 0.003. (0.21 MB TIF) [file pone.0010886.s001.tif]

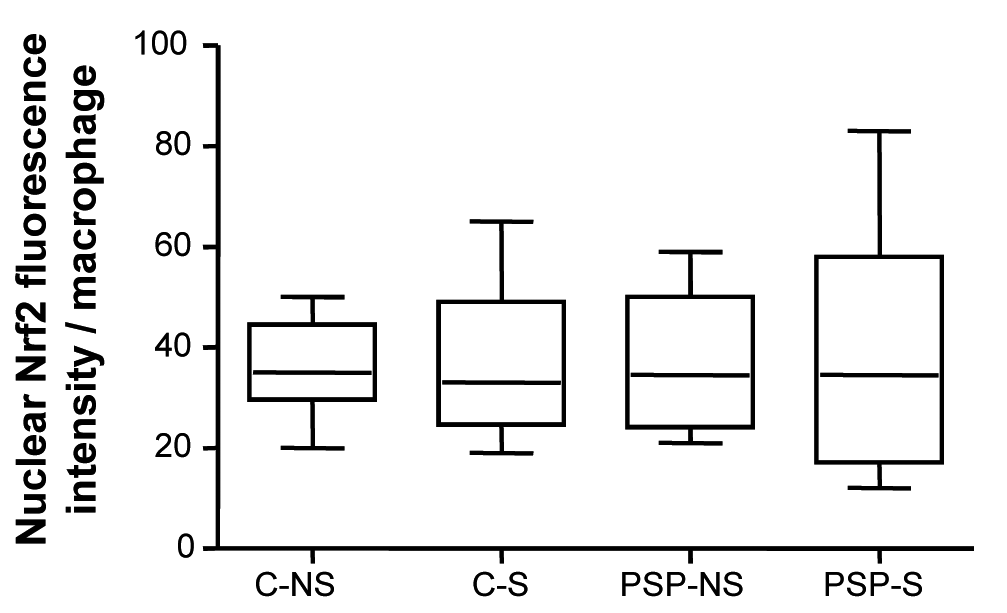

Supplement: Figure S2 — Confocal laser microscopy analysis of Nrf2 expression in lung biopsies. Quantification of nuclear Nrf2 immunofluorescence in macrophages of C-NS, C-S, PSP-NS and PSP-S patients. Abbreviations are in Figure S1. Box-and-whiskers plot with median, interquartile range and minimum and maximum values. (p = 0.99). (0.09 MB TIF) [file pone.0010886.s002.tif]

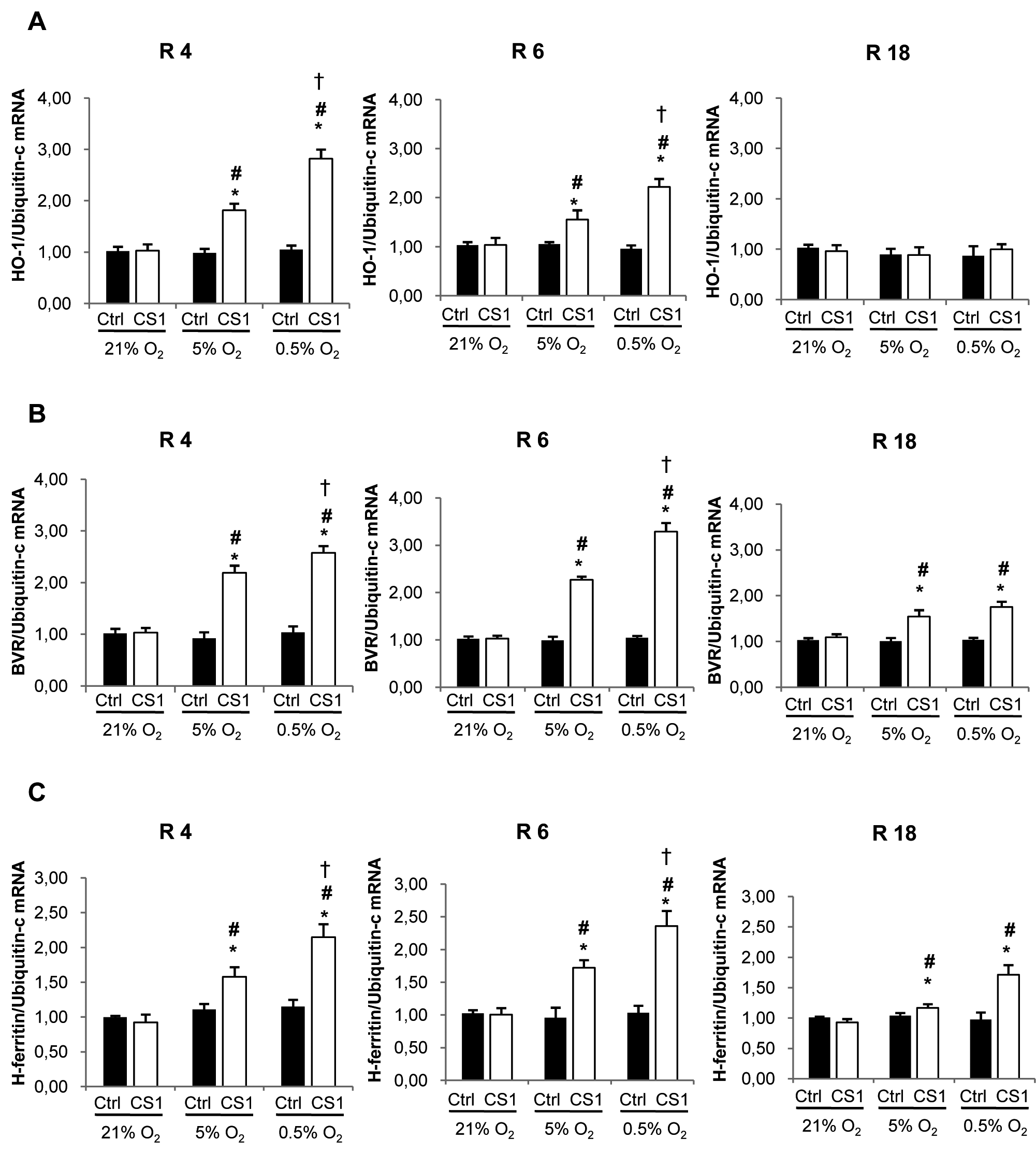

Supplement: Figure S3 — mRNA expression of HO-1 (A), BVR (B) and H-ferritin (C) in THP-1 cells exposed to normoxic (21% O2) or hypoxic (5% and 0.5% O2) conditions and 1 µg/ml CS or DMSO (Control) 4 h (R4), 6 h (R6) and 18 h (R18) after reoxygenation. *p = 0.029 vs. Ctrl, # p = 0.029 vs. 21% O2, + p = 0.029 vs. 5% O2. (0.66 MB TIF) [file pone.0010886.s003.tif]

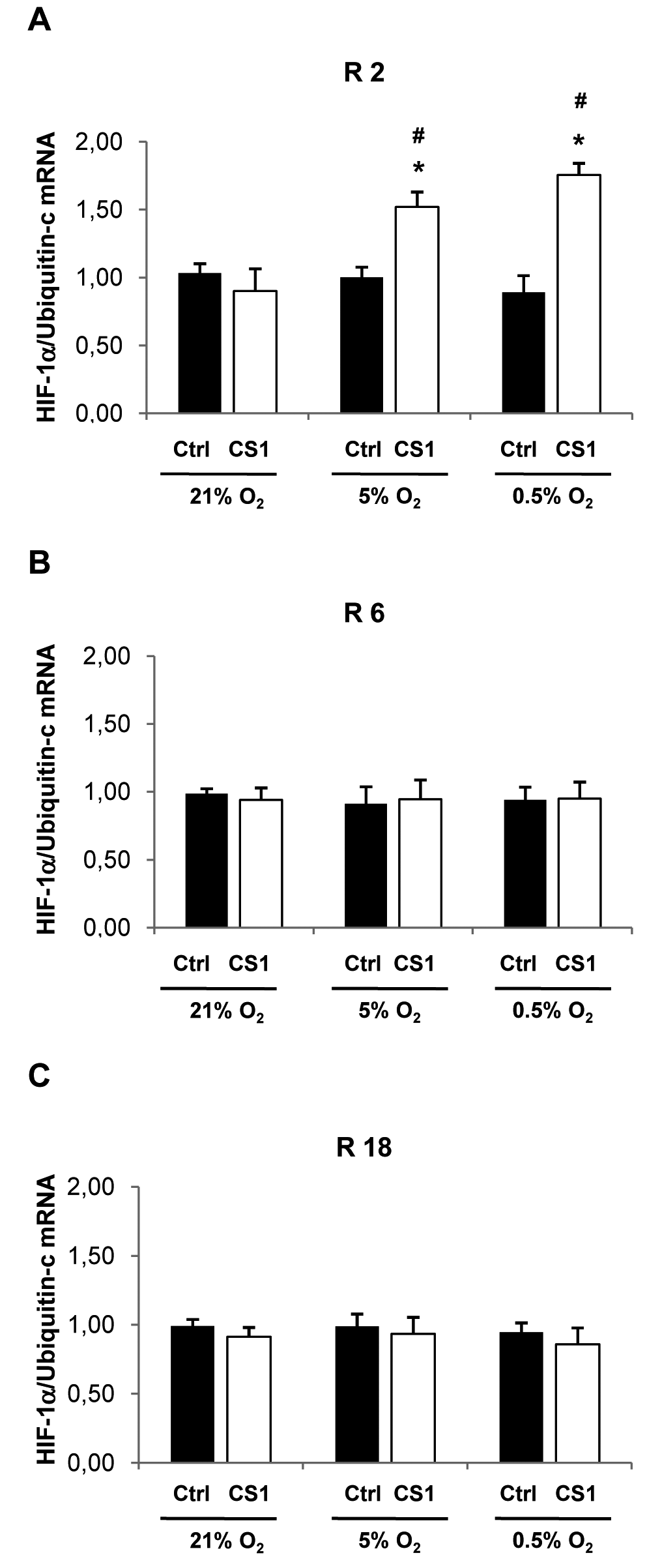

Supplement: Figure S4 — HIF-1alpha mRNA expression in THP-1 cells exposed to normoxic (21% O2) or hypoxic (5% and 0.5% O2) conditions and 1 µg/ml CS or DMSO (Control). Panel A: at 2 h reoxygenation (R2), panel B: at 6 h reoxygenation (R6), panel C: at 18 h reoxygenation (R18). Results are expressed as ratio of expression to that of Ubiquitin-c. *p = 0.029 vs. Ctrl and # p = 0.029 vs. 21% O2. (0.30 MB TIF) [file pone.0010886.s004.tif]
