# Supplementary material for: Induction of Heme Oxygenase-1, Biliverdin Reductase and H-Ferritin in Lung Macrophage in Smokers with Primary Spontaneous Pneumothorax: Role of HIF-1α
Source: PLoS One. 2010 May 28;5(5):e10886. doi: 10.1371/journal.pone.0010886 (PMC2878337; doi:10.1371/journal.pone.0010886)
Supplement: Table S1 — Clinical characteristics of patients. (0.03 MB DOC) [file pone.0010886.s005.doc]

**Table S1:** Clinical characteristics of patients

|  | **C-NS (n=10)** | **C-S (n=9)** | **PSP-NS (n=6)** | **PSP-S (n=9)** | ***p*-value** |
| --- | --- | --- | --- | --- | --- |
| **Age (yrs)** | 56 [19-72] | 57 [30-68] | 26 [19-48] * | 27 [20-40] * | 0.001 |
| **Sex ratio (F/M)** | 1/9 | 0/9 | 1/5 | 1/8 | ns |
| **Smoking (pack.yrs)** | 0 | 35 [10-50] | 0 | 8 [2-30] † | <0.0001 |
| **Current smokers/ex-smokers** | NA | 7/2 | NA | 9/0 |  |
| **Time since smoking cessation (yrs)** | NA | 0 [0-20] | NA | NA |  |

C-NS = control patient non-smokers; C-S = control patient smokers; PSP-NS = primary spontaneous pneumothorax non-smokers; PSP-S = primary spontaneous pneumothorax smokers; yrs = years; F = female; M = male.

Results are presented as median [min-max], * p=0.001 vs. C-NS and C-S; † p<0.0001 vs. C-S as assessed by Mann-Whitney U-test; NA: not applicable; ns: non significant.
